# Mammary gland specific expression of Brk/PTK6 promotes delayed involution and tumor formation associated with activation of p38 MAPK

**DOI:** 10.1186/bcr2946

**Published:** 2011-09-17

**Authors:** Kristopher A Lofgren, Julie H Ostrander, Daniel Housa, Gregory K Hubbard, Alessia Locatelli, Robin L Bliss, Kathryn L Schwertfeger, Carol A Lange

**Affiliations:** 1 Department of Medicine (Division of Hematology, Oncology, and Transplantation), University of Minnesota, 420 Delaware St. SE, MMC 806, Minneapolis, MN 55455, USA; 2 Microbiology, Immunology and Cancer Biology Graduate Program, University of Minnesota, 420 Delaware St. SE, MMC 196, Minneapolis, MN 55455, USA; 3 Masonic Cancer Center, University of Minnesota, 425 E. River Road, Minneapolis, MN 55455, USA; 4 Department of Pathology, Third Faculty of Medicine, Ruska 87, 100 00 Praha 10, Czech Republic; 5 Department of Lab Medicine and Pathology, University of Minnesota, 420 Delaware St SE, MMC 609, Minneapolis, MN 55455, USA; 6 Department of Pharmacology, University of Minnesota, 321 Church St SE, Minneapolis, MN, 55455, USA

## Abstract

**Introduction:**

Protein tyrosine kinases (PTKs) are frequently overexpressed and/or activated in human malignancies, and regulate cancer cell proliferation, cellular survival, and migration. As such, they have become promising molecular targets for new therapies. The non-receptor PTK termed breast tumor kinase (Brk/PTK6) is overexpressed in approximately 86% of human breast tumors. The role of Brk in breast pathology is unclear.

**Methods:**

We expressed a WAP-driven Brk/PTK6 transgene in FVB/n mice, and analyzed mammary glands from wild-type (wt) and transgenic mice after forced weaning. Western blotting and immunohistochemistry (IHC) studies were conducted to visualize markers of mammary gland involution, cell proliferation and apoptosis, as well as Brk, STAT3, and activated p38 mitogen-activated protein kinase (MAPK) in mammary tissues and tumors from WAP-Brk mice. Human (HMEC) or mouse (HC11) mammary epithelial cells were stably or transiently transfected with Brk cDNA to assay p38 MAPK signaling and cell survival in suspension or in response to chemotherapeutic agents.

**Results:**

Brk-transgenic dams exhibited delayed mammary gland involution and aged mice developed infrequent tumors with reduced latency relative to wt mice. Consistent with delayed involution, mammary glands of transgenic animals displayed decreased STAT3 phosphorylation, a marker of early-stage involution. Notably, p38 MAPK, a pro-survival signaling mediator downstream of Brk, was activated in mammary glands of Brk transgenic relative to wt mice. Brk-dependent signaling to p38 MAPK was recapitulated by Brk overexpression in the HC11 murine mammary epithelial cell (MEC) line and human MEC, while Brk knock-down in breast cancer cells blocked EGF-stimulated p38 signaling. Additionally, human or mouse MECs expressing Brk exhibited increased anchorage-independent survival and resistance to doxorubicin. Finally, breast tumor biopsies were subjected to IHC analysis for co-expression of Brk and phospho-p38 MAPK; ductal and lobular carcinomas expressing Brk were significantly more likely to express elevated phospho-p38 MAPK.

**Conclusions:**

These studies illustrate that forced expression of Brk/PTK6 in non-transformed mammary epithelial cells mediates p38 MAPK phosphorylation and promotes increased cellular survival, delayed involution, and latent tumor formation. Brk expression in human breast tumors may contribute to progression by inducing p38-driven pro-survival signaling pathways.

## Introduction

Total protein tyrosine kinase (PTK) activity is elevated in breast cancer [1] and this condition is associated with poor prognosis [2]. PTKs and their downstream signaling pathways contribute to critical biological functions relevant to the cancerous phenotype, such as increased cellular proliferation, pro-survival, invasion and migration/metastasis. One such cancer-associated PTK is breast tumor kinase/protein tyrosine kinase 6 (Brk/PTK6). Brk was cloned in a screen for tyrosine kinases expressed in a metastatic breast tumor [3]. The murine Brk-ortholog, Src-like intestinal kinase (Sik), was independently cloned from the small intestine and skin and found to share 80% identity with Brk [4]. Although considered to be only distantly related to c-Src, Brk shares a similar domain structure, consisting of an N-terminal SH2 domain, an SH3 domain, and a C-terminal kinase domain that is subject to autophosphorylation and autoinhibition [5]. However, the Brk C-terminus lacks a motif required for myristoylation (that is, as found in c-Src), rendering it truly "soluble" or mobile within and between cellular compartments [3,4].

Brk is overexpressed in up to 86% of invasive ductal breast carcinomas [6,7], prostate and colon carcinomas [8,9], 70% of serous ovarian carcinomas [10], 37.5% of a limited sampling of head and neck squamous cell carcinomas [11], and a small percentage of metastatic melanomas [12]. Brk expression levels increase in association with the carcinoma content of breast tumors [7], tumor grade [13], and invasiveness of breast cancer cell lines [14]. Normal tissues that express Brk include the intestinal epithelium, melanocytes, keratinocytes [4,15], prostate luminal epithelium [16], and lymphocytes [17]. However, Brk appears to be absent from normal mammary tissue [8]. The list of Brk substrates and interacting proteins is limited, but consists largely of signaling or signal transduction-related adaptor molecules, and RNA- or DNA-binding proteins, including signal transducers and activators of transcription (STATs). Notably, both STAT3 and STAT5b have been shown to be direct substrates of Brk *in vitro *[18,19]. These molecules are also required regulators of mammary gland lactogenic differentiation (STAT5, [20]) and regression (STAT3, [21]).

Mammary gland development is a highly dynamic and hormonally-driven process; functional glands are not fully mature until early adulthood or pregnancy. Beginning as an invagination of dermal epithelium (that is, in the embryo), the mammary anlage migrates into the mesenchyme, eventually elongating into a rudimentary branched ductal tree [22]. The gland remains at this primitive state until puberty, when the terminal end buds (TEBs) respond to hormonal cues and lead the advancement and secondary branching of the ductal network further into the mammary fat pad resulting in a network of hollow ducts. Outside of fluctuations in secondary branching due to cycling hormonal cues during the estrous cycle [22], further functional differentiation is temporarily halted until pregnancy. Upon pregnancy, a marked increase in ductal branching and alveolar proliferation and differentiation occurs, preparing the gland for lactogenesis.

Once the suckling stimulus of the offspring is removed, involution is initiated. In mouse models, completion of this regressive 10-day process returns the gland to a near virgin state. Post-lactational involution is characterized by events that can be classified into two distinct stages. First, milk stasis and the resulting mechanical stresses initiate a tightly regulated wave of apoptosis in alveolar epithelial cells and their concomitant removal [23], followed by the second stage in which remodeling of the ECM and the expansion of the stromal adipocyte compartment occurs [24]. Mouse models have been extensively used to understand genetic mechanisms of breast cancer biology [25]. Indeed, classical models of human breast oncogene overexpression in the mouse mammary gland demonstrate altered biological processes responsible for proper ductal and alveolar development, as well as modified initiation and execution of glandular involution [26].

In this study, we describe the first transgenic model of mammary gland specific (that is, WAP-promoter-driven) Brk expression. Using newly created Brk-WAP transgenic mice, we studied the physiological process of mammary gland involution to investigate the impact of Brk expression on the survival of mammary luminal epithelium, and altered regulation of pro-survival signaling pathways that may be permissive for mammary tumorigenesis.

## Materials and methods

### Mice and tissues

Transgenic mice expressing the human Brk/PTK6 gene under the control of the whey acidic protein (WAP) promoter were generated by microinjection of a WAP-Brk insert containing the wild-type Brk cDNA under the control of the WAP gene promoter into FVB/n embryos (University of MN Mouse Genetics Laboratory). The Brk cDNA was subcloned into the WKbpAII vector (a kind gift of Dr. Jeff Rosen, Baylor College of Medicine; [27]) using EcoR1 sites within the multiple cloning sequence. Two founders (one female, one male) were identified by PCR screening of tail biopsy DNA, and confirmed by Southern blotting (data not shown). Primer sequences for genotyping transgenic animals (by collection of DNA harvested from tail biopsies) span the Brk coding sequence (sense, 5'-agcgtgcacaagctgatgct-3') and the bovine growth hormone poly-A region of the transgene (antisense, 5'-tctctggctgtctgtctgca-3'). Experiments were conducted under University of Minnesota IACUC approved protocols and NIH guidelines.

### Involution timecourse

Virgin FVB/n or WAP-Brk mice were bred; litters were carried to term and normalized to eight pups upon parturition. Pups were nursed for 10 days at which time the litter was force weaned. Mammary glands were harvested at one day post-weaning; involution Day 1 (INV1), INV4, INV6, INV9, and INV14.

### Whole mounts

Inguinal mammary glands were harvested and fixed, washed with PBS and stained with Carmine Alum. Glands were then dehydrated in graded ethanols, cleared with xylenes, and affixed to slides.

### IHC and differential stains

A Leica Microsystems 1020 automated processor was used to process tissues after fixation. After paraffinization, three- to five-micron thick sections were cut and mounted on slides.

### Imaging and analysis

Digital images were taken of three fields per gland from three glands at 200 × or 400 × total magnification. For epithelial content determination, a grid of 360 boxes was overlaid on 200 × images and boxes containing epithelial cells were counted. For IHC quantification, NIH ImageJ [28] was used with a cell counter plug-in to manually count positively stained mammary epithelial cells vs. total epithelial cells in multiple fields. Annotated regions were drawn on each digital H&E image using a pen tablet (Intuos3, Wacom, Kazo-shi, Saitama, Japan) for area calculations by determining epithelial pixel count relative to the entire gland, and selecting regions of interest for digital IHC analysis. For digital IHC quantification, slides were scanned at 40 × magnification (0.25 microns/pixel) using a whole slide scanner (ScanScope CS, Aperio Technologies, Vista, CA, USA) fitted with a 20x/0.75 Plan Apo objective lens (Olympus, Center Valley, PA, USA). Images were saved in SVS format (Aperio) compressed with JPG2000 at 70% quality and retrieved from a secure server using whole slide image management software (Spectrum, Aperio).

For automated quantification of molecules visualized by IHC, five annotated regions were drawn on each slide using a pen tablet screen (Cintiq 21UX, Wacom, Kazo-shi, Saitama, Japan) on whole slide images viewed at high resolution using the Aperio system's annotation software (ImageScope 10, Aperio).

To detect individual cells in tissue sections, a nuclear cell quantification image analysis algorithm (IHC Nuclear Quantification, Aperio) was trained on control slides by defining the color vectors for the hematoxylin nuclear counterstain and primary positive chromagen DAB, minimum and maximum size for nuclei, and threshold ranges for intensity of nuclear staining. The analysis algorithm was trained to detect nuclei in four intensity ranges for cells with no positive staining, weak positive staining, medium positive staining, and strong positive staining. Analyses were performed on each annotated region using defined settings and nuclear count results were collected from each slide. Data were represented as an H-score [29], which accounts for staining intensity and percentage of positively stained cells. The H-score = (% of 0 intensity staining nuclei*0) + (% of 1 intensity staining nuclei)*1 + (% of 2 intensity staining nuclei)*2 + (% of 3 intensity staining nuclei) *3. Each H-score represents five fields each from three mice per time point.

### Mammary epithelial cell enrichment

Mammary glands were harvested and weighed. Following disruption with scalpels, tissue homogenates were incubated at 37°C in digestion buffer (Ham's F12/DMEM, 2 mg/mL collagenase A (Roche, Indianapolis, IN, USA), 100 U/ml hyaluronidase (Sigma-Aldrich, St. Louis, MO USA)). Digested mammary tissue was pelleted and washed with Ham's F12/DMEM+1% serum three times at 1, 500 rpm, then twice at 800 rpm. Cell pellets were lysed as in [30] with the addition of Roche PhosStop and Complete tablets.

### Immunohistochemistry (IHC)

Formalin fixed paraffin embedded (FFPE) sections of mammary glands were deparaffinized with xylenes, and rehydrated through graded alcohols (70% to 100%). Rehydrated sections were equilibrated in PBS and microwaved in antigen retrieval buffer (10 mM sodium citrate, pH 6.0 for 20 minutes or 1 mM EDTA, pH 8.0 for 10 minutes). Slides were washed with ddH2O then PBS and placed in 3% H_2_O_2 _for 10 minutes to block endogenous peroxidases.

Sections blocked with serum-free protein block (Dako X0909) were incubated overnight at 4°C with primary antibodies diluted in Dako Antibody Diluent (S0809), washed with PBST and incubated in biotinylated secondary antibody (Vector Laboratories Vectastain Elite Kit, PK-101) for 30 minutes at room temperature. Slides were washed, then incubated with Vectastain Elite RTU ABC reagent (PK-7100) and subjected to colorimetric detection with ImmPACT DAB substrate (Vector Laboratories, SK-4105). Antibodies used for IHC are as follows: Brk was purchased from Santa Cruz Biotechnology (Santa Cruz, CA, USA); phospho-p38 mitrogen-activated protein kinase (MAPK), phospho-STAT3, phospho-STAT5, and cleaved caspase 3 were purchased from Cell Signaling (Danvers, MA, USA).

### Growth factors and cell lines

HC11 murine mammary epithelial cells were plated and transfected with pCMV-3X-FL-Brk constructs using FuGene HD (Roche), serum starved post-transfection, and treated with 500 ng/mL prolactin (Sigma). HMEC-Brk and T47D shRNA stable cell lines described previously [7,31] were treated with 25 ng/mL epidermal growth factor (Sigma). Cells were lysed as previously described [30]. Immunoblotting was performed with Brk (Santa Cruz, in-house antibody), total and phospho STAT5 and total and phospho-p38 MAPK (Cell Signaling), and E-cadherin antibodies.

### Anchorage independence

Six-well dishes were coated with PolyHEMA (20 mg/mL in 98% EtOH) and dried in an incubator overnight. Previously described HMEC+Brk cells [31] or HC11 cells transiently transfected with Brk (as above) were plated at a density of 300 K cells per well, and maintained in culture for 48 hr. Cells in suspension were collected, trypsinized and stained with 0.4% Trypan Blue. Viable cells from each sample were counted in triplicate.

### Tissue microarray

A series of human breast tissue samples surgically obtained from healthy women undergoing reduction mammoplasty (*n *= 23), or with pathological conditions including fibroadenoma (*n *= 22), infiltrating ductal carcinoma (*n *= 23) and infiltrating lobular carcinoma (*n *= 23) were made available as FFPE archival material from the Third Medical Faculty (Charles University, Prague, Czech Republic). The original slides were re-evaluated by a pathologist (DH) to confirm the initial pathology diagnosis, and representative tissue blocks were selected for further processing. Informed consent was obtained, and the use of biopsy material for research was approved by the Ethics Committee of the Third Medical Faculty.

Tissue microarrays were constructed from routinely prepared FFPE tissue blocks in parallel, using a manual tissue arrayer TA1 (A Fintajsl, Czech Republic). The representative area of interest was selected on the original glass slide and corresponding area on donor tissue block was inked. Tissue cylinders, 1.6 mm in diameter, were punched from the marked regions of each donor tissue block, and transferred to a recipient block for the array. One hematoxylin and eosin section was made from each block to ensure the presence of tumor regions.

Scoring of positively stained regions was performed with arbitrary establishment of a threshold for positive IHC staining intensity. The scores consist of 0 = no staining relative to no primary controls, 1 = weak diffuse staining, 2 = moderate diffuse staining, 3 = strong diffuse staining. Any focal staining present increased the score by 1 (that is, a weakly diffuse stain with regions of strong focal staining was scored a 2). Only the adenoma or carcinoma compartment was scored, except in the reduction mammoplasty group, where the entire section was analyzed.

### Statistics

Unless otherwise noted, all results are presented as means +/- SEM. Paired t-tests were conducted on IHC quantification and *in vitro *assays. Tumor incidence was compared between WAP-Brk and wt mice using Fisher's exact test. Tumor latency was estimated using Kaplan-Meier methodology and curves compared between WAP-Brk and wt mice using the Wilcoxon test. A chi-squared test was used to compare the association of tumors staining positive for phospho-p38 MAPK with tumors staining positive for Brk. All statistical tests were conducted at a significance level of 0.05.

## Results

### The WAP-Brk transgene is expressed in the mammary gland

**Figure 1:**
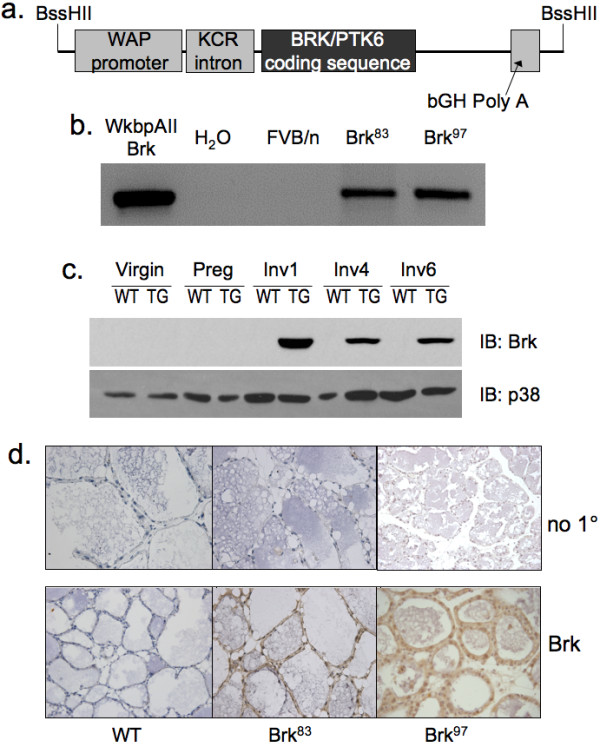
**Transgene, screening and expression**. **A**, Transgene (Tg) structure, including WAP promoter, beta-globin intron to enhance Tg expression, Brk/PTK6 coding sequence, growth hormone poly A signal and BssHII restriction sites. **B**, PCR screening of founder mice and controls. (*L-R*: purified construct, water, wild-type FVB, and two WAP-Brk founders.) **C**, Whole cell lysates from wild type and Brk^97 ^mammary glands were subjected to Western blotting for Brk or total p38 MAPK, after enrichment for epithelial cells. **D**, Brk IHC in FFPE mammary gland sections. Glands shown are from INV D1. Brown precipitate indicates positive staining. 200 × magnification.

To determine the effects of inducible Brk expression in the normal mammary gland *in vivo*, a Brk cDNA encoding the full-length wild-type protein kinase was put under the control of the WAP promoter (Figure 1a), which directs transgene expression predominantly to the luminal epithelium in response to the hormones of pregnancy and upon lactation [32]. Founders were generated and successfully bred to establish two independent lines (Brk^83 ^and Brk^97^); transgene presence was verified by PCR (Figure 1b). Expression of Brk protein was confirmed by both Western blotting of purified mammary epithelial cells (MEC) and IHC analysis of formalin fixed paraffin embedded (FFPE) mammary tissues (Materials and methods). Total p38 MAPK, a ubiquitous member of the MAP kinase family, served as a loading control in Western blotting (that is, of MEC whole cell lysates) experiments. Total p38 levels were somewhat lower in MEC purified from mammary glands of both virgin and pregnant animals, but remained relatively constant during involution Days 1 to 6. In lactating transgenic but not wild-type animals, Brk protein expression was readily detectable at involution Day 1 (lactation Day 11) and this persisted to Day 6 of mammary involution (Figure 1c). Brk expression was significantly reduced (weakly detected) by Day 14 of mammary involution; variable weak expression of Brk also occurred in pregnant transgenic animals, but remained consistently high during lactation (not shown). IHC of FFPE tissues also demonstrated significant Brk protein expression in mammary (luminal) epithelial cells in both transgenic lines (Figure 1d); we designated these independently derived transgenic lines Brk^97 ^(founder #11097) and Brk^83 ^(founder #8383). Brk was undetectable in wild-type controls by both protein detection methods (Figure 1c, d).

### WAP-Brk expression alters the kinetics of mammary gland involution

**Figure 2:**
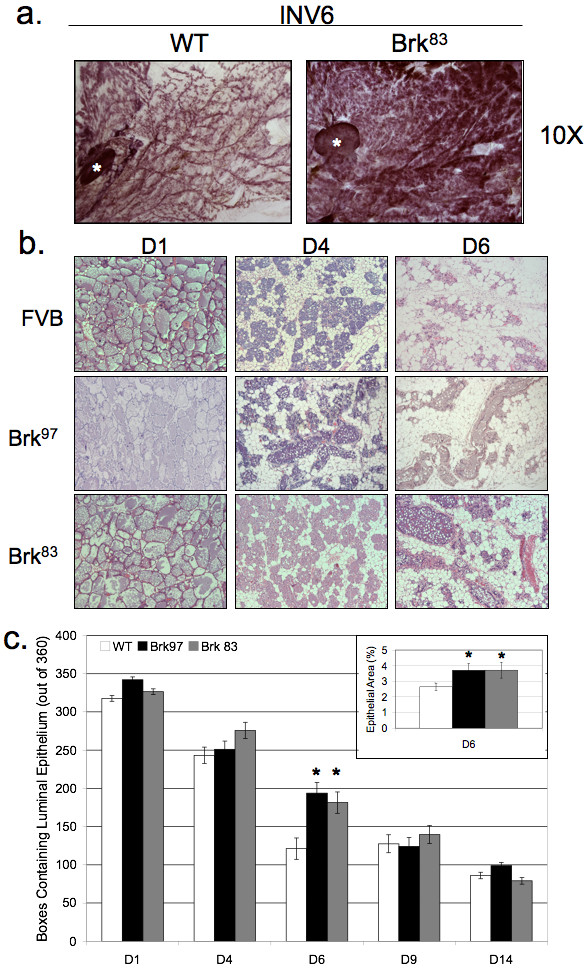
**WAP-Brk mammary glands exhibit delayed post-lactational involution**. **A**, Carmine alum staining of inguinal mammary gland whole mounts, INV D6. **B**, H&E staining of inguinal mammary glands from INV D1-INV D6. 200 × magnification. **C**, Quantification of mammary epithelium from H&E sections, INV D1-D14. *Inset*, epithelial content quantified by area on Day 6. Asterisks signify statistically significant differences between groups (*P *< 0.05).

Based on *in vitro *studies of Brk overexpression in HB4a mammary epithelial cells [33], we hypothesized that Brk may confer a proliferative and/or pro-survival phenotype to normal MEC *in vivo*. Additionally, numerous studies have demonstrated similar phenotypes upon expression of human breast oncogenes in the mouse mammary gland [34,35]. Therefore, we investigated whether Brk expression in the mouse mammary gland resulted in proliferative or inhibitory effects. Upon careful inspection of virgin and pregnant Brk transgenic mice (whole mount analysis), we detected no noticeable differences in mammary gland development (data not shown). However, upon analysis of mammary glands undergoing involution (Days 1 to 6), we noted that glands from Brk transgenic mice were larger and had a higher cellular content than their wild-type counterparts (Figure 2a). Hematoxylin and eosin (H&E) staining of mammary sections revealed a lag in remodeling of glands from Brk transgenic mice relative to wild-type controls harvested at the same stage of forced involution (Figure 2b). Alveoli at Day 1 of involution showed no major differences in development or milk content. However, glands from Brk transgenic mice appeared to have fewer apoptotic epithelial cells being shed into the lumen when compared to matched wild-type animals. Notably, shedding alveolar cells were still present in the lumen on Day 4 of involution in mammary glands of Brk transgenic mice whereas none were present in glands of wild-type mice. Additionally, there appeared to be larger clusters of secretory alveoli in glands of transgenic mice relative to wild-type animals (Day 4). By Day 6, glands of both wild-type and Brk transgenic mice were mostly repopulated with adipocytes, but Brk-transgenic mice still exhibited functional alveoli, as indicated by the noticeable presence of milk and lipid droplets within the luminal spaces; ductal structures were distended and filled with protein and lipid in the transgenic lines, whereas in wild-type mice, ductal structures were collapsed and adipocyte content returned to levels commonly observed for this time point during normal murine mammary gland involution [36]. Days 9 and 14 of involution appeared similar in both wild-type and transgenic lines, consistent with a decline in Brk transgene expression at these late time points (not shown).

In order to quantify the epithelial content of mammary glands from wild-type or Brk transgenic mice, digital images of H&E sections were analyzed [34]. Using an arbitrary grid of 360 boxes overlaid onto an image, the presence or absence of luminal epithelial cells was recorded, and presented as a fraction of the total grid (Figure 2c). Epithelial cell content did not differ during early (Days 1 to 3) involution. However, beginning with Day 4 and significantly at Day 6, glands from Brk transgenic animals presented with higher epithelial content relative to same day wild-type glands; these differences were resolved by Day 9. To validate our analysis, the epithelial cell area was re-calculated by arbitrarily selecting epithelial regions of mammary tissue and determining the area of the selected regions (in pixels); these data are presented as a fraction of the whole gland at Day 6 (inset Figure 2c). Brk^97 ^transgenic mice again demonstrated a statistically significant increase in epithelial cell area when compared to Day 6 wild-type glands (3.6% versus 2.5%, *P *< 0.05). These data suggest that Brk expression induces a delay in mammary gland involution, which is most apparent between Days 4 and 6, and that mammary glands from transgenic mice recover this difference by Days 9 to 14, consistent with the decline in Brk expression by Day 14.

**Figure 3:**
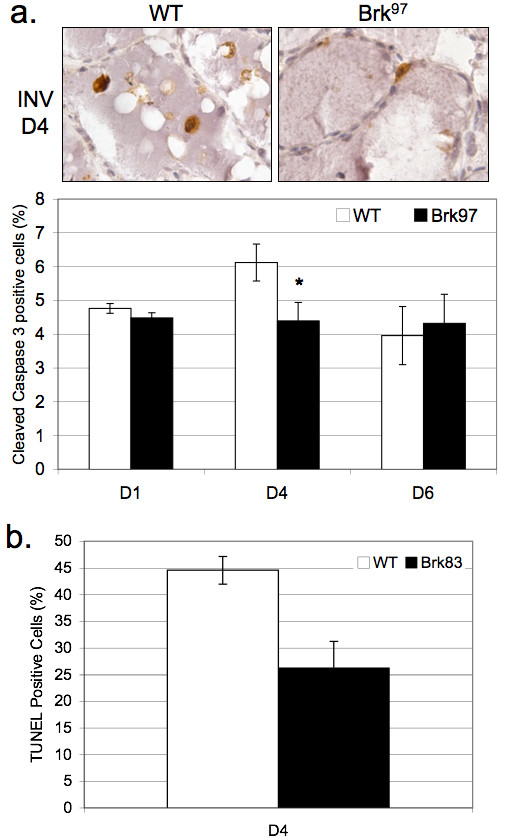
**Apoptotic events are decreased in mammary tissue from WAP-Brk mice**. **A**, Cleaved caspase 3 IHC. *Images*: INV D4 in wild-type and Brk^97 ^mice, 400 × magnification. *Graph*: Quantification of positive IHC staining, presented as a percentage of total mammary epithelium. **B**, Quantification of Terminal deoxynucleotidyl transferase mediated dUTP Nick End Labeling (TUNEL) positive cells in INV D4 mammary gland sections.

To further confirm a Brk-induced delay in mammary gland involution (Figure 2), we investigated signaling inputs associated with early initiation and execution of involution. The first stage of involution (approximately Days 1 to 3) is characterized by massive apoptosis and luminal shedding of epithelial cell bodies formerly lining the ducts [37]. To detect luminal epithelial cells undergoing apoptosis in glands from wild-type or Brk mice, IHC was performed with antibodies directed against cleaved caspase 3. Multiple representative images (Figure 3a, images) were scored for positive cells, with visible dead cells already shed into the lumen also being counted as positive. Data are presented as the percentage of cleaved caspase 3-positive cells relative to total epithelial cells. At Day 4 of involution, fewer cleaved caspase 3-positive cells were present in mammary glands from transgenic mice relative to wild-type controls (Figure 3a, bar graph). By Day 6 of involution, apoptotic cell numbers had reached similar levels in glands from both Brk transgenic and wild-type animals. These results were confirmed by TUNEL staining of apoptotic cells in Day 4 glands (Figure 3b).

**Figure 4:**
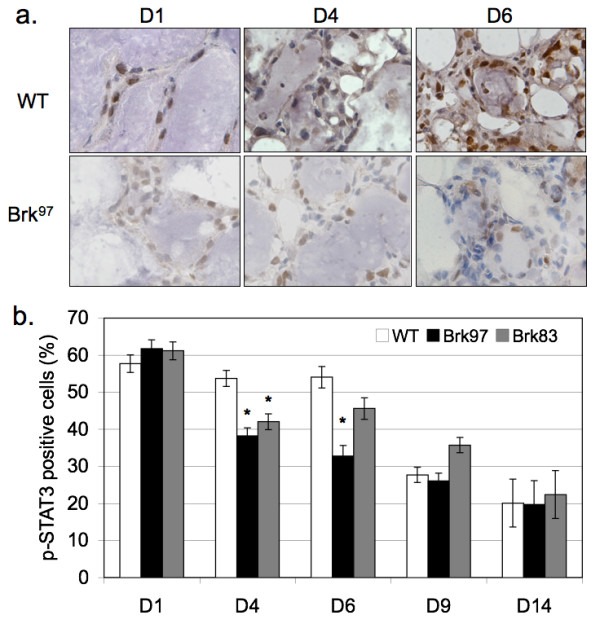
**Involution related STAT3 signaling decreases in WAP-Brk mice**. **A**, Representative phospho-STAT3 IHC in involuting mammary glands from INV D1-INV D6 wild-type and Brk^97 ^mice, 400 × magnification. **B**, Quantification of positive IHC staining, as a percentage of total mammary epithelium. Asterisks signify statistically significant differences between groups (*P *< 0.05).

STAT3 is a critical mediator of the induction of involution [21,38]. Expression of STAT3 is required for mammary involution, and in mouse models, its phosphorylation is induced at the beginning of involution but gradually declines over a six plus day time course [21,38]. To directly measure STAT3 phosphorylation in our Brk transgenic model, IHC was performed on wild-type and Brk-expressing mammary glands during the involution time course. Representative fields (Figure 4a) from each time point were scored for the presence or absence of p-STAT3 in individual cells of the luminal epithelium, and presented as a percentage of total cell count. The levels of p-STAT3 were similar in both lines at involution Days 1 and 14. However, at Days 4 and 6, glands from Brk transgenic animals exhibited roughly 10 to 20% less p-STAT3 relative to controls (Figure 4b). In glands from WAP-Brk mice, the levels of p-STAT3 decreased precipitously from Days 1 to 4, compared to the wild-type mice, in which the decrease was not evident until Day 9. Similar to the epithelial content (Figure 2), STAT3 signaling in glands from transgenic vs. wild-type mice returned to comparable levels after Day 9, resulting in an approximate 20% basal level of STAT3 phosphorylation in resting or fully regressed glands. Taken together, these data support a Brk-dependent delay of early involution, as indicated by fewer apoptotic cells (Day 4); this phenotype is associated with transient suppression of STAT3 phosphorylation (Days 4 to 6), an independent marker of involution induction. As with p-STAT3 (Figure 4b), mammary glands (InvD4) from WAP-Brk transgenic mice contained slightly less p-STAT5 relative to mammary glands from wt mice (not shown).

### Brk expression promotes increased phosphorylation of p38 MAPK

**Figure 5:**
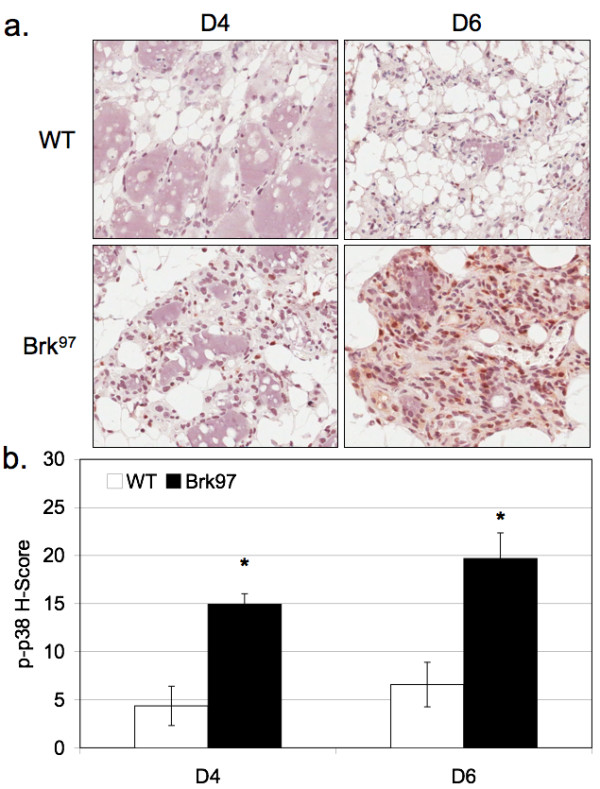
**Brk promotes p38 MAPK activation in mammary glands**. **A**, Phospho-specific p38 MAPK IHC in involuting mammary glands, INV D4-INV D6. **B**, Quantification of positive IHC, presented as an H-score (see Methods).

Previously, we reported that Brk promotes increased breast cancer cell proliferation and migration in response to the erbB ligands, EGF and heregulin, in part via Brk-dependent signaling to p38 MAPK [7]. Although early studies initially described p38 MAPK as a mediator of stress responses and linked to activation of apoptosis pathways in multiple tissues, this kinase is closely associated with pro-survival phenotypes in the context of breast cancer [39]. We predicted that Brk-transgene expression in the mammary gland may increase p38 MAPK activity, perhaps leading to prolonged luminal epithelial cell survival and delayed involution (Figure 2). FFPE sections from involuting mammary glands were processed for phospho-p38 MAPK IHC (Figure 5a), digitally analyzed and assigned H-scores (as above; see Methods); data represent the degree of phospho-p38 MAPK positivity and staining intensity (Figure 5b). Notably, at both Days 4 and 6 of involution, we detected an approximately three-fold increase in the H-scores of glands from Brk transgenic mice relative to glands from same-day wild-type mice. Both the number of cells staining for phospho-p38 and the intensity of staining (per cell) were increased. These data suggest that Brk can drive p38 MAPK signaling *in vivo*; activated p38 MAPK may act as a pro-survival signal in this setting, leading to delayed mammary gland involution.

### Brk-dependent signaling and pro-survival are linked events *in vitro*

**Figure 6:**
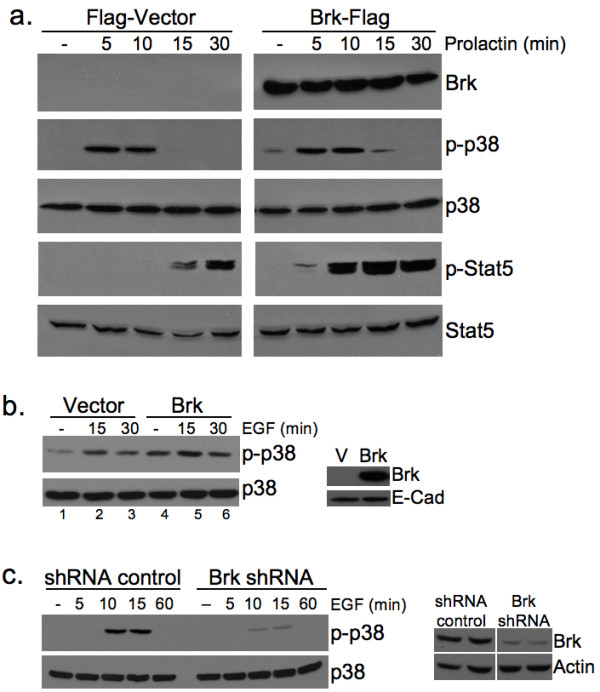
**Brk promotes p38 MAPK signaling *in vitro***. **A**, Brk expression sensitizes HC11 cells to prolactin treatment and increased basal p38 MAPK phosphorylation. Transiently transfected HC11 cells treated with 500 ng/mL prolactin were subjected to Western blotting for STAT5 and p38 MAPK phosphorylation. **B**, Stable expression of Brk in HMEC increases basal p38 MAPK activation. **C**, Brk knockdown in T47D breast cancer cells decreases EGF induced p38 MAPK phosphorylation. Whole cell lysates were subjected to Western blotting for p38 MAPK phosphorylation and/or STAT5 phosphorylation.

To further link Brk expression to activation of p38 MAPK in the mouse model, we introduced flag-tagged Brk (Brk-Flag) or the Flag vector control (Flag) into Sik-null HC11 murine mammary epithelial cells by transient transfection and treated cells with the breast lactogen, prolactin. p38 MAPK is abundantly expressed in these cells. Following a time course of prolactin (500 ng/ml) treatment, Western blotting revealed robust phosphorylation of p38 in both Flag-vector control and Brk-Flag transfected cells (Figure 6a). However, in HC11 cells expressing Brk-Flag, both the intensity (0 to 15 minutes) and kinetics (see 15-minute time point) of p38 phosphorylation were increased relative to vector control cells (Figure 6a). Notably, transient expression of Flag-Brk in HC11 cells also induced increased basal p38 MAPK phosphorylation (that is, weak constitutive phospho-p38 was repeatedly observed in the absence of growth factors and hormones only in Flag-Brk expressing cells). As a control for intact prolactin signaling and Brk-dependent actions, we also blotted for phosphorylated STAT5, a known substrate of activated Brk [19]. Similar to the published results of Weaver and Silva [19] in breast cancer cells, we also observed Brk-mediated regulation of p-STAT5 *in vitro*, but only in prolactin-treated (5 to 30 minutes) cells (Figure 6a). Phospho-STAT3 was not appreciably altered by these treatments (not shown).

To further link Brk overexpression to activation of p38 MAPK, we made use of our previously engineered human mammary epithelial cell (HMEC) lines stably expressing either a vector control or wild-type Brk (HMEC-Brk cells) [31]. Again, Brk expression alone resulted in increased basal phosphorylation of p38 in untreated cells (compare lanes 1 and 4); EGF-induced activation of p38 was also slightly increased upon Brk expression (15 to 30 minutes) relative to vector controls (Figure 6b). Finally, EGF-dependent activation of p38 MAPK was effectively blocked (10 to 15 minutes) upon knock-down (gene silencing) of endogenous Brk in Brk-positive breast cancer cells (Figure 6c).

**Figure 7:**
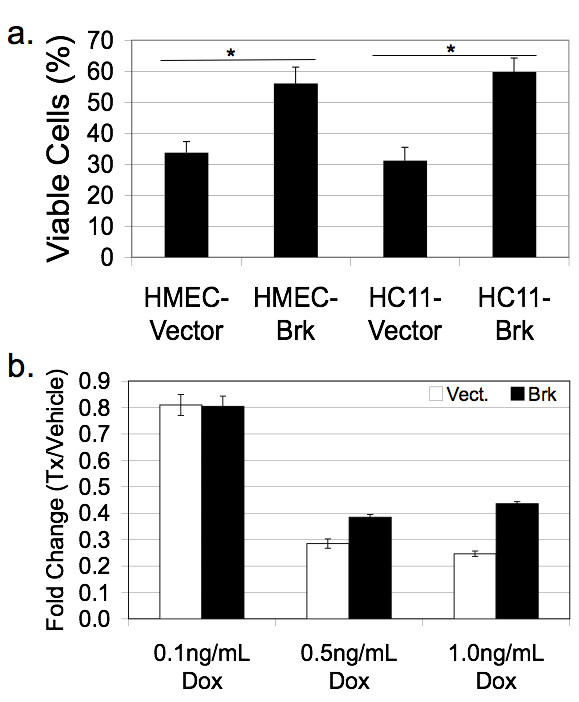
**Brk promotes survival phenotypes *in vitro***. **A**, Exogenous Brk expression in HMEC and HC11 cells promotes anchorage independent survival. **B**, Brk expression in HMEC cells provides partial resistance to doxorubicin. Cells were plated in triplicate in three independent experiments; data shown are from one representative experiment. Error bars represent +/- one standard deviation.

Recent *in vitro *studies have demonstrated that Brk promotes anchorage independent survival [13]. To address the biological effects of Brk overexpression in an additional *in vitro *model of mammary epithelial cells, we again made use of our HMEC lines stably expressing either a vector control or wild-type Brk (HMEC-Brk cells) [31]. These human cell lines and the mouse HC11 model (transiently transfected with vector or Flag-Brk as above) were plated on PolyHEMA, a substrate that coats plastic culture dishes and prevents cell attachment. Following 48 hrs, viable cells in suspension were counted using Trypan Blue dye exclusion. Notably, Brk expression conferred increased anchorage independent survival relative to vector controls in both HMEC and HC11 models (Figure 7a). Interestingly, Brk sensitized non-transformed intestinal epithelial cells to apoptosis [40]. To further test the ability of Brk to alter the survival of MEC, adherent cultures of HMEC cells stably expressing either Brk or control vector were subjected to doxorubicin, a commonly used cytotoxic (DNA damaging) agent. At a dosage of 0.1 ng/ml doxorubicin, we detected no significant difference between HMEC cells expressing Brk and control cells, with approximately 80% of plated cells remaining viable relative to vehicle-treated controls (Figure 7b). However, following 0.5 to 1.0 ng/ml doxorubicin treatment, only 25 to 30% of vector control cells remained viable, whereas approximately 40% of Brk-positive HMEC cells survived this treatment (relative to vehicle controls). These data, together with published findings in breast cancer cell models [13] suggest that Brk expression confers a strong survival signal to both normal and neoplastic mammary epithelial cells.

### WAP-Brk mice develop adenosquamous carcinoma

**Figure 8:**
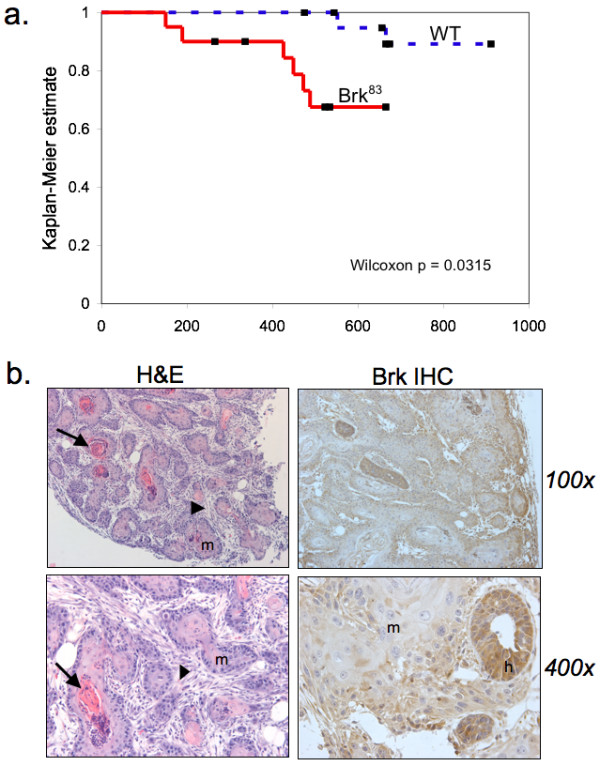
**WAP-Brk mice develop mammary tumors**. **A**, Kaplan-Meier estimate for the tumor cohort desribed in Table 1 (tumor free mice vs. days). **B**, Representative images of a tumor from WAP-Brk mouse B257. Left: H&E sections of a tumor in the R2 mammary gland. *Right*: Transgene expression in the same tumor. (Arrow, keratin pearl; arrowhead: fibrotic stroma; h, hyperplastic duct; m, squamous metaplasia.)

Numerous *in vitro *studies suggest that Brk acts as a breast oncogene. However, this body of work has relied heavily on RNAi approaches primarily performed in transformed and tumorigenic Brk-positive breast cancer cell lines. Experiments expressing Brk in non-transformed mammary epithelial cells usually include cooperating factors. For example, Kamalati *et al. *[33] demonstrated Brk-induced soft-agar colony formation in the EGFR-high cell line HB4a. Coexpression of Brk and ErbB2 in human (MCF-10A) or murine (Comma-1D) mammary epithelial cells induced acinar disruption and increased tumor growth, respectively [41]. To determine if Brk expression acts as a potential singular oncogenic insult in the mammary gland, we aged retired, multiparous WAP-Brk and wild-type mice. Notably, mammary tumors were infrequent events, but developed in multiparous WAP-Brk mice at a three-fold higher incidence relative to wild-type mice (Table 1); this trend failed to reach statistical significance. However, the tumor latency significantly decreased (Table 1 and Figure 8a; Wilcoxon *P *= 0.03). The pathology of mammary tumors arising in Brk-transgenic mice consisted of duct-like structures with squamous metaplasia filling the lumens, surrounded by fibrotic stroma (Figure 8b). Keratin pearls were often present (Figure 8b, arrows) in lesions where the squamous metaplasia predominated. Large pleomorphic nuclei were evident in these regions. Hyperplastic ducts were also detected in nearby regions of otherwise normal tissue architecture (Figure 8b). Brk transgene expression was detected in mammary tumors with strong cytoplasmic staining and sporadic nuclear staining in hyperplastic ducts; weak cytoplasmic staining occurred in cells that had undergone squamous metaplasia (Figure 8b).

**Table 1:** Tumor incidence in wild type vs.

Mouse line	Tumor incidence	Mean age of tumor bearing mice (days)	Mean parity of tumor bearing mice
Wild Type	0.095 (2/21)	608 (552, 665)	3 litters (3)
Brk^83^	0.300 (6/20)	316 (range: 150 to 453)	1.5 litters (range: 0 to 4)

transgenic mice

### Brk signaling intermediates are evident in human breast biopsies

**Figure 9:**
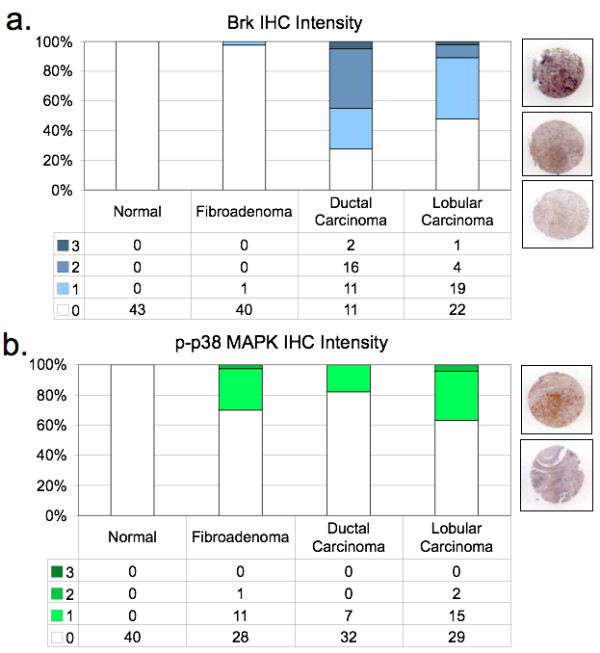
**Brk-mediated signaling events are present in a human tissue array**. **A**, Brk IHC in a human TMA. **B**, Phospho-p38 MAPK IHC in a human TMA. *Graphs*: IHC intensity plotted as a fraction of all tissue samples on the array, totaling 100%. *Images*: Representative images of IHC intensities in carcinoma samples. **C**, Brk and p-p38 MAPK co-staining in human TMA samples. Brk-positive tumors are 2.87 times more likely to be phospho-p38-positive than Brk-null tumors are likely to be phospho-p38-positive (*P *= 0.03). The 95% CI for the odds ratio is (1.10, 7.43).

Although studies are limited, Brk appears to be expressed in a majority of human breast cancers [6,7], and is often co-expressed [42] or co-amplified [41] with Her2. Using a human breast tumor tissue array, we examined Brk co-expression with phospho-38 MAPK in IHC-stained sections of normal breast tissue from reduction mammoplasty (normal breast), fibroadenoma, and infiltrating ductal or lobular carcinoma. Positively-stained sections were further scored for intensity (by pathological criteria) as defined in Materials and methods (Figure 9). Brk protein expression (Figure 9a) was not detected in normal tissue samples (0/43), and occurred in only one of 41 fibroadenoma samples. However, we detected Brk in 72.5% (29/40) of ductal carcinoma samples and 52.2% (24/46) of lobular carcinomas included in the array. These figures are in agreement with our previous report of Brk expression in up to 86% of mammary carcinoma (of 250 samples; [7]). Similar to Brk expression, phospho-p38 MAPK (Figure 9b) was absent from normal breast tissue. However, at least 30% (12/40) of fibroadenoma, 17.9% of ductal samples (7/39), and 37% of lobular carcinoma samples (17/46) stained positively for phospho-p38 MAPK. Representative images of staining intensities appear next to each bar graph for each antibody used in the IHC analysis (Figure 9a, b).

We next examined the frequency of co-staining for Brk and p-p38 within the same samples (Table 2). A significant proportion of ductal and lobular carcinoma samples that stained positive for Brk also co-stained with phospho-p38 MAPK; Brk-positive tumors are 2.87 times more likely to be phospho-p38-positive than Brk-null tumors are likely to be phospho-p38-positive (*P *= .03). These data suggest that Brk is significantly associated with activated p38 in breast cancer relative to normal breast. Brk signaling to p38 MAPK may characterize a subset (approximately 14 to 15%) of human ductal and lobular breast carcinoma. More studies are needed to fully understand the contribution of these pathways (that is, Brk and p38 MAPK in combination) to the development and maintenance or progression of human breast carcinoma.

**Table 2:** Brk co-expression with p-p38 MAPK in human TMA samples

Tissue type	Normal	Fibroadenoma	Ductal carcinoma	Lobular carcinoma
**Brk and p-p38 double positive**	0/35	0/39	7/38	7/46

## Discussion

Herein, we report the first transgenic mouse model of mammary specific (inducible) Brk expression (Figure 1). We illustrate a delay in mammary gland involution following forced weaning (Figure 2). We detected evidence of Brk-mediated signaling through increased phospho-p38 MAPK (Days 4 and 6; Figure 5). Brk expression also partially prevented anoikis in non-transformed HMEC and HC11 cell lines *in vitro *(Figure 7a). Aged, multiparous WAP-Brk mice exhibited a trend towards higher tumor incidence and significantly decreased tumor latency relative to wild-type mice (Table 1); these tumors were Brk-positive (Figure 8b). Finally, we detected significant association of phospho-p38 MAPK in biopsies of Brk-positive human breast cancer (Figure 9 and Table 2). Our studies suggest that Brk confers p38-associated pro-survival signals to non-transformed (luminal) mammary epithelium. Given time, these events may conspire to induce or permit the formation of latent mammary tumors (Figures 8 and 9).

### Involution

Mouse mammary gland involution represents a highly sensitive read-out of human oncogenic action; numerous breast oncogenes induce delayed involution in mouse models [26]. Similar to other models of mammary oncogene expression [34,35], our model undergoes delayed, but ultimately complete mammary regression, highlighting a distinct window of mammary signaling events (Days 4 to 6 of involution) that are perturbed without completely halting the involution process. We observed fewer apoptotic figures, decreased caspase3 cleavage, and reduced TUNEL staining in glands from WAP-Brk transgenic mice, while clearance of apoptotic mammary epithelial cells did not appear to be affected. Notably, WAP-Brk transgenic mice eventually undergo complete mammary regression, consistent with the decline of WAP-driven Brk expression over the time course of involution in this model. Upon multiple rounds of parity induced mammary expansion and contraction, amplified survival signaling may increase the chances for mammary epithelial cells to encounter and fix potentially oncogenic combinatorial events.

### Tumor biology (in vivo model and human tumors)

Inducible Brk expression in our WAP-driven transgenic model results in a tumorigenesis rate of 30% in aged multiparous mice (Table 1 and Figure 8). Two wild-type FVB mice from the same litter also developed tumors. Indeed, this strain has a weak propensity to develop adenosquamous mammary tumors at an advanced age [43]. Because of the sibling wild-type FVB tumors, the comparison of the number of tumors between wild-type and Brk transgenic animals did not reach statistical significance (*P *= 0.13). However, the age at tumor onset decreased (Table 1) and this reduced tumor latency was significantly different from wild-type controls (*P *= 0.03), indicating an effect of Brk expression on the promotion of tumorigenesis relative to wild-type FVB mice. Brk strongly promotes breast cancer cell proliferation [7,44], survival [13] and migration *in vitro *[7,14,44,45]. We did not observe pulmonary metastatic lesions in tumor-bearing WAP-Brk mice, suggesting that other cooperating factors are necessary for invasion and migration *in vivo*. We are currently crossing WAP-Brk mice with other mouse models of breast cancer in order to identify additional oncogenic events that may cooperate with Brk overexpression.

Brk protein is readily detectable in hyperplastic regions of WAP-Brk mammary tumors (Figure 8b). The loss of Brk protein in regions of squamous metaplasia of WAP-Brk tumors is likely due to the loss of mammary epithelial differentiation, an event(s) that may ultimately lead to silencing the WAP promoter. Note that Brk expression may drive the appearance of the squamous metaplasia phenotype directly, as Brk expression in the skin increases during the maturation of keratinocytes, promoting squamous differentiation of the epidermis [46].

Brk appears to predominantly mediate cellular survival/resistance to involution-associated apoptosis in this model. This phenotype is consistent with Brk-dependent activation of p38 MAPK [7], as measured by its increased phosphorylation (Figures 5 and 6). Elevated phospho-p38 (Days 4 and 6) was detected in our involution time course experiments, *in vitro *experiments with Brk-expressing HC11 cells, and in Brk-positive tumors derived from both WAP-Brk mice (not shown) and humans (Table 2 and Figure 9b). As expected, IHC analysis of the human breast tumor tissue array revealed Brk expression in only one (a fibroadenoma) of the 84 non-transformed tissue samples (Figure 9a), and in Brk-positive tumors, Brk expression was significantly associated with increased phospho-p38 (Table 2); the samples in this group were mostly derived from premenopausal women.

### Brk mediated signals

The separation of normal physiological cues from transgene-mediated signaling is critical to understanding events that may contribute to mammary oncogenesis. Initial characterization of WAP-Brk mammary glands focused on STAT3 signaling as a marker of mammary gland involution. STAT3 is a required mediator of involution-related cell death [21], and has been reported to be a Brk substrate in studies using cell lines [18]. Our IHC analysis illustrated that glands of WAP-Brk mice contain less p-STAT3 during involution (Days 4 and 6) relative to wild-type animals (Figure 4). These results suggest that while Brk mediated STAT3 phosphorylation may be relevant to the growth of established mammary tumors (that is, breast cancer cells [47]), forced expression of Brk does not appear to drive phospho-STAT3 during the initiation of involution. Similar to p-STAT3, Brk expression *in vivo *also suppressed early (Day 4) p-STAT5 levels; rapid loss of STAT5 phosphorylation, characteristic of involution, occurred in both wt and transgenic animals (data not shown). Thus, Brk does not appear to be a positive regulator of STAT3 or STAT5 *in vivo*; STAT3 phosphorylation may serve primarily as an indicator of the progress of mammary involution herein. Other factors (not addressed herein) that may contribute to Brk-dependent pro-survival include amplification of signaling pathways downstream of erbB family members [33,48], including activation of ERK5 signaling [7,45]. These pathways are frequently associated with breast cancer progression and invasive tumor behavior [49,50].

We have previously described Brk mediated p38 MAPK signaling as primarily promoting cell migration in EGF or heregulin-treated breast cancer cell lines [7]. However, there are limited studies investigating the role of p38 MAPK activity in mouse models of breast cancer. Demidov *et al. *[51] expressed an MMTV-driven active MKK6 (an upstream kinase in the p38 MAPK module), and showed resistance to development of ErbB2 *and *Wip1 induced mammary tumors; however, when overexpressed, MKK6 may regulate other MAPKs [52]. Similar to our studies, Leung *et al. *[35] expressed MMTV-V12Rac3 and described incomplete involution associated with elevated phospho-p38 MAPK. Additionally, Wang *et al. *[53] overexpressed activated Pak1 under the β-lactoglobin promoter and reported a 20% tumorigenesis rate and elevated phospho-p38 MAPK. In both of these studies, as well as ours, there were detectable levels of phospho-p38 in wild-type cohorts, strongly suggesting an as of yet under appreciated physiological role for p38 MAPK in mammary gland biology. Mammary glands from WAP-Brk transgenic mice exhibited higher phospho-p38 levels relative to wild-type glands during Days 4 and 6 of the involution time course (Figure 5), again consistent with an increased survival stimulus in WAP-Brk mice. Interestingly, expression of Brk in HC11 or HMEC cells increased basal phospho-p38 in serum-starved cells (Figure 6), indicating that the presence of Brk is sufficient to promote p38 MAPK activation and survival of mammary epithelium. These data suggest that p38 phosphorylation induced by Brk expression in non-transformed mammary epithelium could contribute to breast disease as either an early event (allowing pro-survival and/or luminal filling) or late event (migration/dissemination, therapy resistance) in tumorigenesis, thereby leading to a poor prognosis. Recent literature [13] and our observations in HMEC and HC11 cell lines (Figure 7) illustrate that Brk promotes anchorage independent survival. Importantly, this has been shown to be a p38 MAPK-dependent phenotype in Brk-positive MDA-MB-468 cells [54]. Taken together, these data suggest that Brk-mediated p38 activation is likely a critical node for mammary epithelial cell pro-survival and relevant to early oncogenic signaling; p38 inhibitors may present an opportunity for therapeutic intervention aimed at long term breast cancer prevention and/or increased sensitivity to chemotherapeutic agents.

## Conclusions

This study characterizes the first mouse model of mammary gland specific Brk/PTK6 expression, and identifies Brk-dependent signaling pathways associated with pro-survival (p38 MAPK). Brk/PTK6 expression in non-transformed mammary epithelium causes delayed involution and promotes early tumorigenesis in aged mice, with signaling that recapitulates the same altered signaling pathways present in human tumor biopsies. The identification of Brk-dependent signaling events reveals potential therapeutic targets (Brk, p38 MAPK) for Brk-positive breast cancers.

## Abbreviations

Brk: breast tumor kinase; DOX: doxorubicin; ECM: extracellular matrix; EGF: epidermal growth factor; FFPE: formalin-fixed: paraffin-embedded; H&E: hematoxylin and eosin; HMEC: human mammary epithelial cells; IHC: immunohistochemistry; INV: involution; MAPK: mitrogen-activated protein kinase; MEC: mammary epithelial cells; PBS: phosphate buffered saline; PTK: protein tyrosine kinase; SH2: Src-homology 2 domain; SH3: Src-homology 3 domain; Sik: Src-like intestinal kinase; STAT: signal transducer and activator of transcription; TMA: tissue microarray; WAP: whey acidic protein.

## Competing interests

The authors declare that they have no competing interests.

## Authors' contributions

KAL, KLS and CAL designed the experiments. KAL and GKH purified mammary epithelium, while KAL, DH and GKH performed IHC and histology. KAL, JHO and AL performed *in vitro *experiments. DH prepared breast tumor tissue arrays, and KAL and DH analyzed pathology data. RB performed statistical analysis on animals and TMA studies. KAL and CAL wrote the manuscript.
